# Assessment of Overactive Bladder after Laparoscopic Lateral Suspension for Pelvic Organ Prolapse

**DOI:** 10.1155/2019/9051963

**Published:** 2019-04-04

**Authors:** Ewelina Malanowska, Andrzej Starczewski, Włodzimierz Bielewicz, Matteo Balzarro

**Affiliations:** ^1^Department of Gynaecology, Endocrinology and Gynaecologic Oncology of Pomeranian Medical University, Szczecin 70-001, Poland; ^2^Department of Urology, Azienda Ospedaliera Universitaria Integrata of Verona, 37121 Verona, Italy

## Abstract

**Background:**

Pelvic organ prolapses (POP) and overactive bladder (OAB) may coexist and both negatively impact quality of life in women. The correlation between POP and OAB remains unclear, but these patients may have the OAB resolution after the surgical treatment of POP. Aim of our study was to assess the anatomical results and the effect on OAB symptoms in women who underwent laparoscopic lateral suspension for POP.

**Materials and Methods:**

This prospective study included all women with apical POP who underwent surgical repair with laparoscopic uterine lateral suspension from January 2016 to December 2017. The baseline and the 1-year follow-up included post-void residual measurement, urinalysis, vaginal examination, OAB symptoms evaluation, and administration of questionnaires (PFDI-20, UDI 6).

**Results:**

64 women underwent laparoscopic lateral suspension for uterine prolapse and 78.1% had concomitant anterior vaginal wall defect. At 1-year follow-up the anatomic success rates were 84.4% for the apical and 76.2% for the anterior compartment. The comparison between OAB symptoms before and after the surgical procedure showed the resolution of OAB in 76% of the women, while de novo OAB was present in 2.6%. With the questionnaires 95.3% (61/64) of our patients were satisfied after the POP repair. We documented a trend in ameliorating of OAB regardless of the POP-Q stage. However, the Pearson test showed this correlation as statistically significant only in women with anterior vaginal wall defect stage III and apical stage II. No patient had vaginal exposure of the polypropylene mesh.

**Conclusion:**

Our data show how laparoscopic lateral suspension is an effective procedure for apical and anterior vaginal wall defects. This study provides further evidence for the concept that OAB in women with POP >II stage improves after a successful POP surgery. These women may benefit from a resolution of OAB and POP symptoms with the improvement of patient's quality of life.

## 1. Introduction

Pelvic organ prolapses (POP) are one of the most common indications to surgery due to their detrimental effect on the quality of life [1, 2]. Overactive bladder (OAB) is also a disturbing common condition defined as the urinary urgency, usually accompanied by increased urinary frequency and nocturia, with or without urgency urinary incontinence, in the absence of urinary tract infection or other obvious pathology [3].

POP and OAB symptoms are frequently encountered in the same patient [4]. The correlation between POP and OAB remains unclear. A potential cause of OAB may result from mechanical bladder outlet obstruction (BOO) [5]. In Urology it is well-known how in males the benign prostatic hyperplasia may create a chronic bladder obstruction resulting in OAB symptoms [6]. With a similar mechanism a POP may have an obstructive action on the female urethra creating the base to develop OAB symptoms. Indeed, patients may present a spectrum of voiding complaints and symptoms of both OAB and BOO. POP repair usually resolves the mechanical BOO but the effect on OAB symptoms may be unpredictable [7]. However, despite these hypotheses, the relationship between POP and OAB is not clear. If there is a causal relationship it could be anticipated that OAB symptoms would improve after successful treatment of POP [5]. Therefore, POP surgery may cure or improve OAB, or it can result in de novo OAB [8–10].

Focusing on the upper vaginal compartment prolapses, the vaginal vault prolapse, or the uterine prolapse, several authors reported the effect of the different surgical techniques on OAB. In one RCT, Halaska et al. reported new OAB symptoms after vaginal vault repair with sacrospinous fixation or transvaginal mesh ranging from 9% to 21% [11]. Maher et al. reported de novo OAB after sacrospinous fixation and sacrocolpopexy, respectively, at 20.6% and 33.3% [12]. The first aim of this study was to assess the anatomical results and the effect on OAB symptoms in a cohort of women who underwent laparoscopic lateral suspension for POP.

## 2. Material and Methods

The study was approved by the Ethics Committee on Clinical Studies of Pomeranian Medical University. This prospective study included all women consecutively referred to our Department from January 2016 to December 2017, with symptomatic apical prolapse who underwent primary surgical repair with laparoscopic uterine lateral suspension.

Objective evaluations were performed with the International Pelvic Organ Prolapse Staging System (POP-Q), and the prolapse was assessed by maximum Valsalva effort in the seated semi-lithotomy position. Subjective assessment was achieved by the Pelvic Floor Distress Inventory Questionnaire (PFDI-20) and Urogenital Distress Inventory 6 (UDI 6).

We offered all patients comprehensive preoperative patient-centered counselling providing them with information and allowing them to participate in the decision-making process as reported in the recent literature [13]. Exclusion criteria were post-void residual volume, posterior vaginal wall defects, previous prolapse or incontinence surgeries, previous hysterectomy, neurological conditions, uncontrolled diabetes, and bladder pain syndrome. Stress urinary incontinence was not an exclusion criterion, but patients were informed that only surgical repair of POP would be done.

All surgical procedures were performed by a senior skilled surgeon (WB). Prophylactic antibiotics were routinely administered intravenously before surgery with 1 g cefazolin i.v. All patients were given low-molecular-weight heparin prophylaxis.

Follow-up was scheduled 12 months after the surgery and performed by a skilled urogynecologist (EM). Objective cure was defined in case of POP-Q sites Ba, C, and Bp as less than -1 cm stage at any point in time of follow-up. Pelvic floor disorders, lower urinary tract symptoms, and digestive symptoms were detailedly recorded. Tract urinary infection was excluded by urinalysis, and trans-vaginal ultrasonography was performed to assess the post-void residual urine evaluation.

OAB was assessed by response to (i) UDI 6 item number 1, (ii) UDI6 item number 2, and (iii) the interview at the follow-up. De novo SUI was assessed by UDI 6 item 3, and stress test.

The use of drugs affecting OAB was investigated and recorded.

Data were entered into the database by one author (EM) and double-checked by another author (AS). Complications were reported according to Clavien-Dindo classification (reference).

### 2.1. Surgical Technique

All women underwent laparoscopic supracervical hysterectomy. A T-shaped polypropylene mesh was used for the lateral suspension. The body of mesh was fixed to the uterine cervix and to the upper part of the anterior vaginal wall. The arms were introduced retroperitoneally towards lateral abdominal walls, alongside round ligaments. After the prolapse reduction using a posterior blade of speculum placed in the anterior vaginal fornix the mesh was tension-free suspended.

### 2.2. Statistical Evaluation

Data analysis was performed using the Student t-test, Pearson's correlation, and Gretl Software ver. 2017a. P value less than 0.001 was considered statistically significant.

## 3. Results

Sixty-four women who had uterine prolapse were consecutively included in the study, 78.1% of these (50/64) had a concomitant anterior vaginal wall defect, and no patient was lost at the follow-up. Demographic characteristics of the population are reported in Table 1.

A mild SUI was present in 21.8% of the population. These women reported the use of no more than one small pad/day.

All surgical procedures were done under general anesthesia. In 2/64 women (3.1%) there was a bladder injury that was resolved intraoperatively by suturing and leaving the urinary catheter for 7 days, rated grade 1 on the Clavien-Dindo classification. Operating time varied between 90 and 260 minutes depending on the number of surgical steps. No associated surgical procedure was done, and no blood transfusion was required. Patients were discharged from the hospital after 4-5 days. No woman had post-void residual requiring clean intermittent catheterization, or indwelling catheter at the discharge from the hospital. Postsurgical pain control was obtained with paracetamol, and no patient required more than 2 days of therapy.

At one-year follow-up the anatomic success rates were 84.4% (54/64) for the apical compartment, and 76.2% (32/42) for the anterior compartment. De novo posterior vaginal wall defect was present in 4.7% (3/64) of the population: one patient developed an enterocele in (1.6%), and two a rectocele (3.1%) (Table 5). The comparison between objective evaluation before and after the surgical procedure is reported in Table 2, whereas symptoms before and after surgery are listed in Table 3.

With the questionnaires 95.3% (61/64) of our patients were satisfied after the POP repair also in case of POP recurrence due to its lower stage at the POP-Q, and 4.7% (3/64) were dissatisfied with the procedure due to a POP recurrence stage like it was before the surgical treatment.

**Figure 1:**
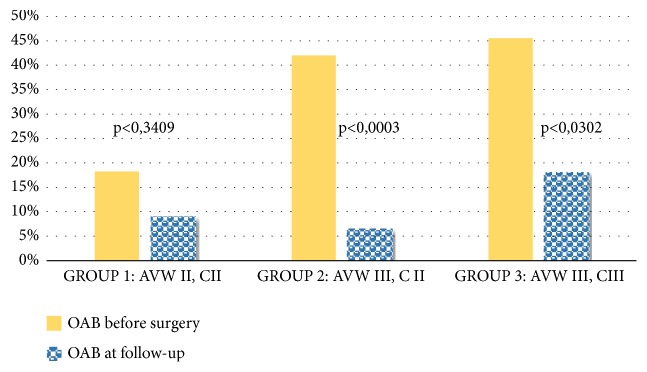
Subdivision of the cohort in three groups according to POP stage and correlation between stages and overactive bladder symptoms before surgery and at the 1-year follow-up. AVW*, anterior vaginal wall*; C,* cervix*; II*, II*°*stage* POP-Q; III,* III*°*stage* POP-Q.

Subjective evaluation showed how 76% of the patients with preoperative OAB had the resolution of symptoms, while de novo OAB was present in 2.6% (Table 4). No patient was under therapy for OAB. To make a correlation between the different stages of POP and OAB, we divided the population into three groups: (i) Group 1 was composed of 11 women with anterior vaginal wall and cervix defect, both stage II; (ii) Group 2 was composed of 31 women with anterior vaginal wall defect stage III and cervix defect stage II; (iii) Group 3 was composed of 22 women with anterior vaginal wall and cervix defect, both stage III. This subanalysis documented a trend in ameliorating of OAB regardless of the POP-Q stage. However, the Pearson correlation showed this correlation as statistically significant only in women of Group II due to the low sample size of Groups I and III (Figure 1). No patient had vaginal exposure of the polypropylene mesh, or complained of urinary tract infections.

Table 5 reports the recurrence and reoperation rates. In this table the two women with postoperatively recurrent cystoceles had a predominant anterior vaginal wall prolapsed (Ba>C) as compared to those with uterine prolapse only (C>Ba).

**Figure 2:**
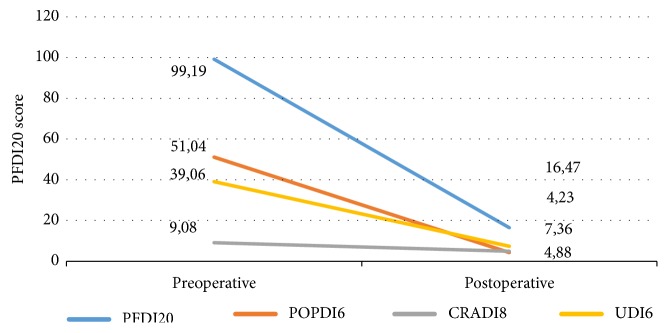
Representation of change before and after surgical treatment at the questionnaires. PFDI20,* Pelvic Floor Distress Inventory*, POPDI6,* Pelvic Organ Prolapse Distress Inventory*, CRADI6,* Colorectal-anal distress inventory*, UDI6,* Urinary Distress Inventory*.

The analysis of validated questionnaires showed the improvement of symptoms and quality of life as reported in Table 6 and represented in Figure 2.

## 4. Discussion

In our study data show how laparoscopic lateral suspension with mesh is a feasible and safe technique with good anatomic results at one-year follow-up. Moreover, we documented a strong coexistence of OAB among patients with anterior vaginal wall defect and/or apical >2 stage II POP-Q. Before the surgical POP repair 39.1% of the women had OAB with 60.9% reporting urinary frequency ≥8/day, and 43.7% of nocturia. In the current literature the higher incidence of OAB in women with POP is well-known varying between 37 and 50% [14–16]. This provides some epidemiological evidence for the concept that anatomic defect may entail OAB symptoms [17]. Interestingly the prevalence of OAB was greater in women with higher stage POP-Q, but its cure was statistically significant only in anterior vaginal wall stage III and cervix stage II POP. Liedl et al. identified the higher prevalence of OAB in stage 2 POP than those in stages III-IV [18]. Our data showed similar results with a trend of improvement in all the stages of POP but achieving a significant improvement after POP treatment only in anterior vaginal wall stage III and cervix II. These findings confirm also what was reported by Petros who recognized OAB symptoms in Half Way System classification low grade POP and the cure of OAB after POP surgical treatment [19].

Our results support the conclusions of previous studies, which determined that OAB may improve, and even resolve, after successful POP surgery [14, 20–22]. OAB and symptomatic POP negatively impact the quality of life of women. However, these patients with the surgical treatment of POP seem also to have the resolution of OAB. The finding of our investigation is that adequate pelvic floor surgery can resolve OAB.

Considering stress urinary incontinence we documented a 64.3% of resolution probably due to the pre-op mild SUI requiring no more than one small pad/day in the patients who used it. The data of persistent SUI in 1/3 of the patients suggest that a concomitant SUI procedure should be proposed after appropriate counselling. Surprisingly an accurate physical examination did not eliminate the appearance of occult stress urinary incontinence (de novo SUI). The use of an accurate counselling was extremely useful and probably helped to improve the approval of the surgical procedure.

A first limitation of our study was dividing the population into 3 groups to correlate the different POP stages with OAB; we did not gain a sample size, in Groups 1 and 3, able to establish the statistically significant improvement. However, the trends are all in the ameliorating direction and bigger numbers would confirm this data.

A second potential limitation is the 1-year follow-up that would have been better if it had been longer. Nevertheless, it should be considered that 12 months is more than enough time to evaluate the evolution of OAB in patients surgically treated for POP, and it is a sufficient time to evaluate anatomical POP results. Moreover, with a follow-up of 1-year we were able not to lose patients.

Women with POP complain of a vaginal bulge or pressure, but they often report other coexisting pelvic symptoms that affect urinary function. The absence of a bulge during a postoperative pelvic examination does not accurately reflect postoperative patient satisfaction, and the presence of an asymptomatic POP recurrence without bladder symptoms does not necessarily correlate to an unsatisfied patient. For these reasons symptoms affecting bladder function, like OAB, should be investigated before and after the surgical POP repair.

Our study suggests that the surgical treatment of apical descensus and cystocele by laparoscopic lateral suspension resulted in the significant improvement in prolapse, OAB symptoms, and patients' quality of life.

## 5. Conclusion

Our data show how laparoscopic lateral suspension is an effective procedure for apical and anterior vaginal wall defects. This study provides further evidence for the concept that OAB in women with POP >II stage significantly improves after a successful POP surgery. These women may benefit from a resolution of OAB and POP symptoms with the improvement of patient's quality of life.

## Figures and Tables

**Table 1:** Patients' characteristics.

Demographics	*n *= 64	
Age (years), mean (±SD)	59.4	(±9.3)
Menopausal status, n (%)	60.0	(93.7)
Systemic HRT at the time of surgery, n (%)	10.0	(15.6)
Body mass index, mean (±SD)	26.8	(±3.5)
Number of vaginal deliveries, mean (±SD)	2.17	(±1.1)
Birth weight of largest baby (g), mean (±SD)	3.74	(±421)
Age at the menopause (years), (±SD)	49.9	(±4.2)
Only uterine prolapse (%)	14	(21.9)
Uterine and anterior vaginal wall prolapse (%)	50	(78.1)

*HRT*: hormone replacement therapy; *SD*:standard deviation.

**Table 2:** Objective assessment: preoperative and the follow-up.

POP-Q parameters	Preoperative	Follow-up at 12 months	*P *
	*Mean *(SD)	*Mean* (SD)	
Aa	0.80 (±0.95)	-1.69 (±0,89)	<0,001
Ba	1.67 (±1,13)	-1.63 (±1,11)	<0,001
C	-0.06(1.63)	-5.55(±2,53)	<0,001
GH	4.00 (±0.59)	2.77 (±0.75)	<0.001
PB	2.33 (±0.84)	2.66 (±0.62)	<0.006
TVL	10 (±0)	10 (±0)	-
Ap	-0.44 (±1.08)	-1.54(±1,04)	<0,001
Bp	-0.47 (±1,01)	-2.19 (±2,29)	<0,001

*POP-Q*, Pelvic Organ Prolapse Quantification System.

**Table 3:** Symptoms before surgery, and at the 12-month follow-up.

	Preoperative *n* (%)	Follow-up *n *(%)	Valuable positive change, *n* (%)	Valuable negative change, *n* (%)	*P *
Bulging	62/64 (96.9)	10/64 (15.6)	52/62 (83.9)	0/2 (0.0)	<0,001
UUI	26/64 (40.6)	10/64 (15.6)	18/26 (69.2)	2/38 (5.3)	<0,001
SUI	14/64 (21.9)	7/64 (10.9)	9/14 (64.3)	2/50 (4.0)	<0,042
Urinary frequency*∗*	39/64 (60.9)	13/64 (20.3)	27/39 (69.2)	1/25 (4.0)	<0,001
Nocturia (≥ 1)	28/64 (43.7)	1/64 (1.6)	27/28 (96.4)	-	<0,001
Overactive Bladder	25/64 (39.1)	7/64 (10.9)	19/25 (76.0)	1/39 (2.6)	<0,001
Constipation	39/64 (60.9)	22/64 (34.4)	18/39 (46.1)	1/25 (4.0)	<0,001
Sexual activity	37/64 (57.8)	38/64 (59.4)	3/27 (11.1)	2/37 (5.4)	<0,658

*UUI,* urgency urinary incontinence; *SUI*, stress urinary incontinence, *∗* > 8 times/day.

**Table 4:** Overactive bladder symptoms, and stress urinary incontinence at the 12-month follow-up.

	*n *	%
Overactive bladder symptoms		
Resolution	19/25	76.0
Persistence	6/25	24.0
De novo	1/39	2.6
Stress urinary incontinence		
Resolution	9/14	64.3
Persistence	5/14	35.7
De novo	2/50	4.0

**Table 5:** Recurrences and reoperation rates.

	*n*	%
Total recurrences	8	12.5
Anterior vaginal wall recurrences	2	3.1
Apical recurrences	3	4.7
Enterocele recurrence	1	1.6
Posterior vaginal wall recurrences	2	3,1
Need for reoperation	7	10.9

**Table 6:** Subjective changes measured by validated questionnaires before and after the surgical treatment.

Questionnaires	Preoperative		Postoperative		*P *
	*mean *	*SD *	*mean *	*SD *	
PFDI20	99.2	± 33.4	16.5	±21.6	<0,001
POPDI6	51.0	± 18.3	4.2	±10.7	<0,001
CRADI8	9.1	± 9.8	4.8	±7.6	<0,001
UDI6	39.1	± 22.3	7.4	±13.3	<0,001

PFDI20, *Pelvic Floor Distress Inventory*; POPDI6, *Pelvic Organ Prolapse Distress Inventory*; CRADI6, *Colorectal-anal distress inventory*; UDI6, *Urinary Distress Inventory*.

## Data Availability

The data used to support the findings of this study are included within the article.
